# Temperature Drift Compensation for Four-Mass Vibration MEMS Gyroscope Based on EMD and Hybrid Filtering Fusion Method

**DOI:** 10.3390/mi14050971

**Published:** 2023-04-28

**Authors:** Zhong Li, Yuchen Cui, Yikuan Gu, Guodong Wang, Jian Yang, Kai Chen, Huiliang Cao

**Affiliations:** 1 Shanxi Software Engineering Technology Research Center, Taiyuan 030051, China; 2 School of Software, North University of China, Taiyuan 030051, China; 3 School of Instrument and Electronics, North University of China, Taiyuan 030051, China; 4 Beijing Institute of Aerospace Control Devices, Beijing 100039, China; 5 School of Automation Engineering, University of Electronic Science and Technology of China, Chengdu 611731, China; 6 Key Laboratory of Instrumentation Science & Dynamic Measurement, Ministry of Education, North University of China, Taiyuan 030051, China

**Keywords:** four-mass vibration MEMS gyroscope, theoretical simulation, temperature drift compensation, empirical mode decomposition, radial basis function neural network, genetic algorithm, Kalman filter

## Abstract

This paper presents an improved empirical modal decomposition (EMD) method to eliminate the influence of the external environment, accurately compensate for the temperature drift of MEMS gyroscopes, and improve their accuracy. This new fusion algorithm combines empirical mode decomposition (EMD), a radial basis function neural network (RBF NN), a genetic algorithm (GA), and a Kalman filter (KF). First, the working principle of a newly designed four-mass vibration MEMS gyroscope (FMVMG) structure is given. The specific dimensions of the FMVMG are also given through calculation. Second, finite element analysis is carried out. The simulation results show that the FMVMG has two working modes: a driving mode and a sensing mode. The resonant frequency of the driving mode is 30,740 Hz, and the resonant frequency of the sensing mode is 30,886 Hz. The frequency separation between the two modes is 146 Hz. Moreover, a temperature experiment is performed to record the output value of the FMVMG, and the proposed fusion algorithm is used to analyse and optimise the output value of the FMVMG. The processing results show that the EMD-based RBF NN+GA+KF fusion algorithm can compensate for the temperature drift of the FMVMG effectively. The final result indicates that the random walk is reduced from 99.608°/h/Hz^1/2^ to 0.967814°/h/Hz^1/2^, and the bias stability is decreased from 34.66°/h to 3.589°/h. This result shows that the algorithm has strong adaptability to temperature changes, and its performance is significantly better than that of an RBF NN and EMD in compensating for the FMVMG temperature drift and eliminating the effect of temperature changes.

## 1. Introduction

MEMS (micro-electro-mechanical systems) gyroscopes are widely used in both civil and military fields. They are mainly used in fields such as automobile navigation, inertial navigation, attitude determination, robotics, micro-signal detection, aerospace and spatial positioning, and application electronics [1,2,3,4,5,6]. However, temperature variation can seriously reduce the precision of the MEMS gyroscope when used in a changing environment. Therefore, it is necessary to study how to eliminate the influence of temperature drift on MEMS gyroscopes. In general, there are two main methods for the temperature compensation of MEMS gyroscopes: hardware compensation methods and software compensation methods.

The hardware method is mainly used to optimise the structure of the MEMS gyroscope. For example, Yu proposed to use a polarising resonator to improve the thermal stability of a resonator fibre optic gyro [7]. Gao proposed that by calculating the optimal layer number of the fibre coil, the temperature drift of the interferometric fibre optic gyroscope can be effectively reduced [8]. Wang proposed that the use of a symmetrical push-pull structure can eliminate the effects of temperature drift [9]. Yan proposed to use a single-polarisation fibre, and the resonant fibre optic gyro was found to exhibit good stability over a wide temperature range [10]. Ling proposed a novel winding idea of dicyclic fibre coils for suppressing the thermal-induced bias drift error of interferometric fibre optic gyroscopes [11]. Qian proposed a hybrid resonator consisting of a polymer-based long-range surface plasmon polariton (LRSPP) waveguide coupler and a silica fibre to reduce the effects of temperature noise on the resonant fibre optic gyroscope [12]. Zhang proposed an improved QAD fibre coil, which helped to reduce the thermal-induced drift error of a fibre optic gyroscope and improved its precision [13]. Zhang proposed a special resonator structure that can generate oriented thermal stress to reduce the temperature drift of the vibrating axis of the resonator [14]. To some extent, hardware compensation methods have the disadvantage of increasing the size and power consumption and cost of MEMS gyroscopes, so these methods are not suitable for some applications that require low cost and low power.

The second solution is a software compensation method that takes as its input the relationship between the temperature of the MEMS gyroscope and the output signal and then establishes a temperature drift model to compensate for the effects of temperature drift. For example, Fontanella proposed a new method for accurate thermal compensation of the inertial device gyro bias using an augmented state Kalman filter [15]. Cao proposed a fusion algorithm based on an RBF NN-GA-KF, which effectively reduced the effect of the temperature drift on the MEMS vibration gyroscope [16]. Baxpameeb proposed an improved method for real-time thermal drift compensation of fibre optic gyros, through which the fibre gyro temperature drift was effectively compensated [17]. Narasimhappa proposed an ARMA model based on an adaptive unscented fading Kalman filter to reduce the drift of a fibre-optic gyroscope [18]. Zhang proposed a novel wavelet threshold denoising method, which effectively suppresses the environmental drift of fibre optic gyroscopes [19]. Song proposed a new modelling and compensation method combining an artificial fish swarm algorithm (AFSA) and a back-propagation (BP) neural network to improve the output precision of fibre optic gyroscopes [20]. Han proposed a simplified model to compensate for the output of ring laser gyros, which effectively improved the output accuracy of the gyroscopes [21]. Ahtohoba used factors such as the temperature change rate of the environment; an error model of the fibre optic gyroscope was established, and the output precision of the gyro was ultimately improved [22]. Zha established an IUKF neural network model for temperature drift compensation of the fibre optic gyro [23]. Prikhodko proposed to compensate high-Q MEMS gyroscopes by using temperature self-sensing technology, and the compensated gyroscope has high precision [24]. Chen proposed a fibre optic gyro adaptive positive linear prediction denoising algorithm based on the ambient temperature rate of change and a temperature drift modelling compensation concept, which was used to correct errors caused by drastic changes in the ambient temperature [25]. Wang proposed to establish an HRG temperature compensation model based on the natural frequency of the resonator and then used this model to compensate for the temperature drift of the gyroscope; this method can be used over a wide temperature range [26]. Cai established a parallel processing model based on multi-objective particle swarm optimisation based on a variational modal decomposition-time-frequency peak filter (MOVMD-TFPF) and Beetle antennae search algorithm-Elman neural network (BAS-Elman NN) to eliminate the noise and temperature drift in a Micro-Electro-Mechanical Systems (MEMS) gyroscope’s output signal [27]. Zhou proposed the improved VMD and TFPF hybrid denoising algorithm, which combines variational modal decomposition (VMD) and time-frequency peak filtering (TFPF), which has a smaller signal distortion and stronger denoising ability [28]. In recent years, the academic circle has shown great interest in the research of a four-mass gyroscope. Zhou proposed a novel Center Support Quadruple Mass Gyroscope, which combines the advantages of tuning fork gyros and micro hemisphere resonant gyros and is expected to achieve performance breakthroughs of flat structures [29]. Trusov proposed a new tuning fork architecture addressing the limitations of the conventional designs [30]. The software method has the advantages of a simple structure, low cost, and convenient implementation, and it is convenient for improving the precision of the MEMS gyroscope.

In this paper, an improved RBF NN filtering algorithm based on EMD is proposed, and this method has been successfully applied to eliminate the temperature drift of the MEMS gyro. Different from the other MEMS gyroscope temperature compensation algorithms, this paper proposes a new fusion algorithm (EMD-based RBF NN+GA+KF fusion algorithm) to eliminate the influence of temperature drift on MEMS gyroscopes and make the MEMS gyroscope more accurate.

The structure of the MEMS gyroscope is introduced in Section 2; the fusion algorithm is shown in Section 3; Section 4 describes the temperature experiment; and Section 5 gives the results of the data processing and a comparison of the various algorithms. The conclusion of this paper is given in Section 6.

## 2. Structure of the Four-Mass Vibration MEMS Gyroscope

**Figure 1:**
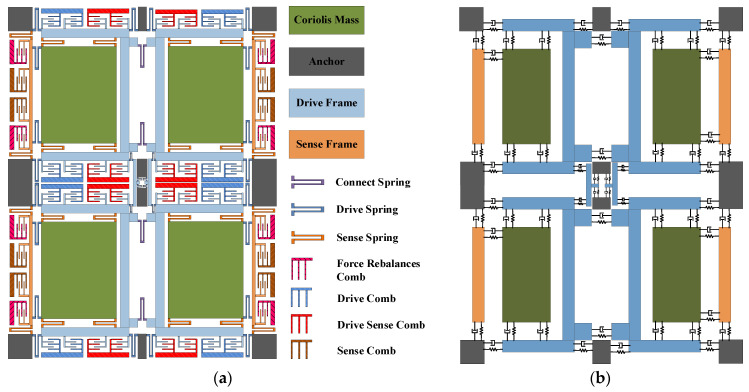
(**a**) Structure of FMVMG, (**b**) The lumped model of FMVMG.

The original signal is generated by a four-mass vibration MEMS gyroscope (FMVMG). The schematic diagram of the FMVMG is shown in Figure 1a. The FMVMG is composed of support anchor points, quadruple mass blocks, a drive comb, a drive sense comb, a sense comb, a force rebalances comb, and multiple folding beams. This gyroscope has two operating modes: a drive mode and a sense mode. Both modes can be considered as a “mass-spring-damped” second-order vibration system. The basic mechanical equivalent model is shown in Figure 1b.

**Figure 2:**
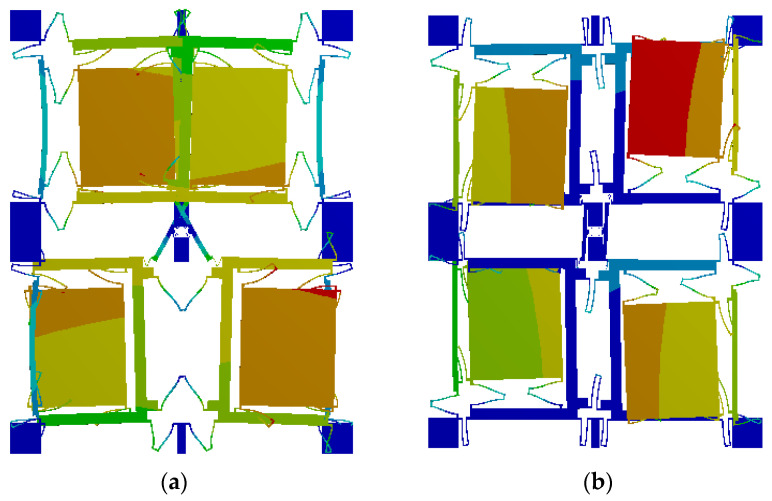
(**a**) Driving mode, (**b**) Sensing mode.

Firstly, after the preliminary design and optimisation, the geometric shape of the FMVMG was determined, and the specific parameters are shown in Table 1. Then, the modal component in ANSYS software was used to analyse the modal of the FMVMG, and the modal analysis mainly included the natural frequency, modal shape, and vibration stability of the MEMS gyroscope structure. Finally, the driving and sensing mode shapes of the FMVMG are shown in Figure 2.

As shown in Figure 2, the vibration mode of the driving mode is the motion of the Coriolis mass block on the *X*-axis, while the vibration mode of the sensing mode is the motion of the Coriolis mass block on the *Y*-axis. In both the driving and sensing modes, the motion of the adjacent mass blocks is reversed. Among them, the resonant frequency of the driving mode is 30,740 Hz, and the resonant frequency of the sensing mode is 30,886 Hz due to the highest mechanical sensitivity when the natural frequencies of the driving and sensing modes of the MEMS gyroscope are equal, but the bandwidth is small. The frequency difference of this structure is 146Hz, which not only ensures the mechanical sensitivity of the gyroscope but also gives it a certain bandwidth.

**Figure 3:**
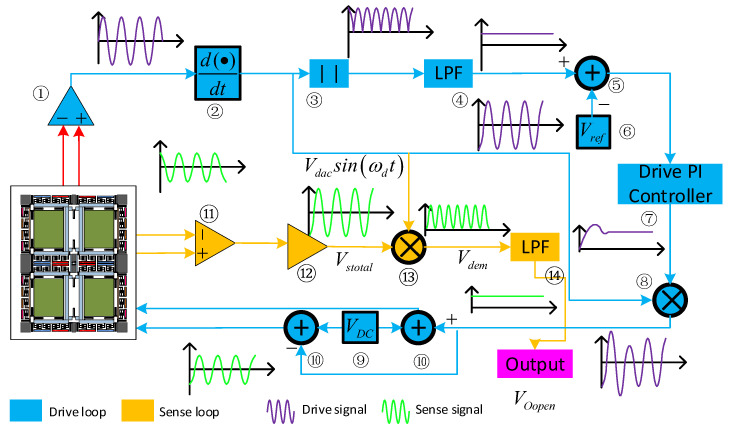
FMVMG monitoring system.

The gyroscope control and detection system are shown in Figure 3. In the drive loop, the drive frame displacement, *x*(*t*), is detected by the drive sensing combs and picked up by the differential amplifier ①. Then, the signal phase is delayed by 90° (through ②) to satisfy the phase requirement of the AC drive signal, *V_dac_Sin(ω_d_t)*. After that, *V_dac_Sin(ω_d_t)* is processed by a full-wave rectifier ③ and a low pass filter ④. Afterwards, *V_dac_* is compared (in ⑤) with the reference voltage, *V_ref_* ⑥. Next, the drive PI controller ⑦ generates the control signal, which is modulated by *V_dac_Sin(ω_d_t)*, and then the signal is superposed (through ⑩) by *V_DC_* ⑨ to stimulation drive mode.

The sensing system employs an open loop, which utilises the same interface as the drive circuit. Firstly, the left and right masses’ sensing signals are detected separately with the differential detection amplifier ⑪. In addition, the output signals are processed by a second differential amplifier ⑫ to generate a signal, *V_stotal_*. Then, *V_stotal_* is demodulated by signal *V_dac_Sin(ω_d_t)* (in ⑬). After that, the demodulated signal, *V_dem_*_,_ passes through the low pass filter ⑭ so the sensing mode’s movement signal, *V_Oopen_*_,_ can be obtained.

**Figure 4:**
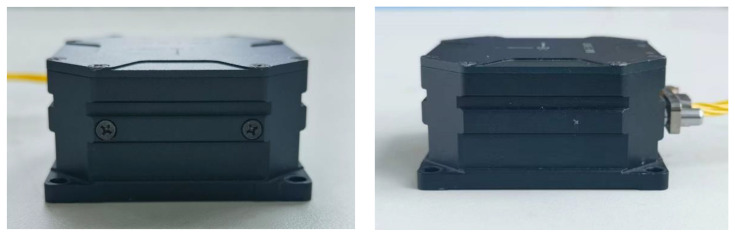
FMVMG prototype.

The monitoring system consists of three PCBs. PCBI contains the connection circuit and is connected to the structure chip, and PCB II is the drive circuit. PCB I and PCB III are nested on the top and back sides, respectively. PCB III contains the detection loop, and the output signal is from PCB III. The prototype of the FMVMG is shown in Figure 4.

## 3. Model and Algorithm

### 3.1. SE-EMD Algorithm

Empirical mode decomposition is a new adaptive signal time-frequency processing method that is especially suitable for the analysis and processing of nonlinear non-stationary signals. In essence, this method is also a process of smoothing non-stationary signals by generating a series of data sequences, and their feature scales are different. Each of these data sequences is called an intrinsic mode function (IMF). The specific steps of the EMD are as follows:

(1) Take out all the local extremum points of the signal and use the cubic spline curve to connect all the local maxima to form the upper envelope. Similarly, the minimum values are also connected to form the lower envelope. In addition, find the average value of the upper and lower envelopes, denoted as *m*_1_, and *h*_1_ can be obtained as follows:

(1) $$x(t)-m_{1}=h_{1}$$

where *x*(*t*) is the original data, and if *h*_1_ meets the IMF component conditions, then *h*_1_ can be seen as the first IMF component of *x*(*t*).

(2) If *h*_1_ is not the IMF, set *h*_1_ as the original data, continue with step 1, obtain the average value *m*_11_, and evaluate *h*_11_ = *h*_1_ − *m*_11_; if *h*_11_ is still not an IMF component, repeat the loop *k* times and obtain *h*_1(*k*−1)_ − *m*_1*k*_ = *h*_1*k*_ until *h_*1*k_* meets the conditions of the IMF. Note that *c_*1*_ = h_*1*k_*. Then, *c*_1_ is the first of the signals *x*(*t*) that meets the IMF condition.

(3) Separate *c*_1_ from *x*(*t*) and obtain Equation (2):

(2) $$r_{1}=x(t)-c_{1}$$

(4) Substitute the value solved by Equation (2) into Step 1 and Step 2, obtain the second IMF-compliant component, and repeat this step *n* times to obtain the *n*th IMF-compliant components of the signal, namely:

(3) $$\left\{\begin{array}{c} r_{1}-c_{2}=r_{2}\\ \cdot \cdot \cdot \\ r_{n-1}-c_{n}=r_{n} \end{array}\right.$$

(5) If the resulting *r_n_* is a monotonic function and no additional IMF-compliant components can be derived from it, the steps are not repeated. The final result of EMD decomposition is shown in Equation (4):

(4) $$x(t)=\sum_{i=1}^{n}c_{i}+r_{n}$$

where *c*_1_, *c*_2_, *c*_3_, …, *c_n_* are the individual IMF components, and *r_n_* represents the average trend of the signal.

After EMD decomposition, the output signal of the gyroscope is decomposed into many IMF components. To simplify the analysis, the IMFs are divided into three component types: a noise component, a mixed component, and a drift component. The sample entropy (*SE*) is introduced here to realise this work. The sample entropy is a measure of the complexity of a time series. The larger the value of *SE* is, the more complex and irregular the data. *SE* can be defined as follows:

(5) $$SE(m,r)=\underset{N\to \infty }{\lim}\{-ln[\frac{A^{m}r}{B^{m}r}]\}$$

where *m* is the embedding dimension, referred to as the length of the data to be compared, and *r* is the tolerance value.

### 3.2. The Algorithm of RBF NN+GA+KF

The radial basis function (RBF) is a non-negative nonlinear function characterised by a local distribution of the centre point and a symmetric attenuation of the radial centre point. The smaller the distance between the input point and the centre of the radial basis function, the stronger the output signal.

The basis function of the RBF NN is shown in Equation (6):

(6) $$\alpha_{j}(x)=\psi_{j}(\left\|x-c_{j}\right\|/\sigma_{j})$$

where *x* is an n-dimensional input vector, *c_j_* is the centre of the jth hidden node, *σ_j_* is the breadth of the hidden layer neuron, ||***_·_***|| is the Euclidean distance, and *ψ_j_* has a maximum value at *c_j_*, which is a radially symmetric function. When ||*x−c_j_*|| increases, *ψ_j_* decreases quickly.

**Figure 5:**
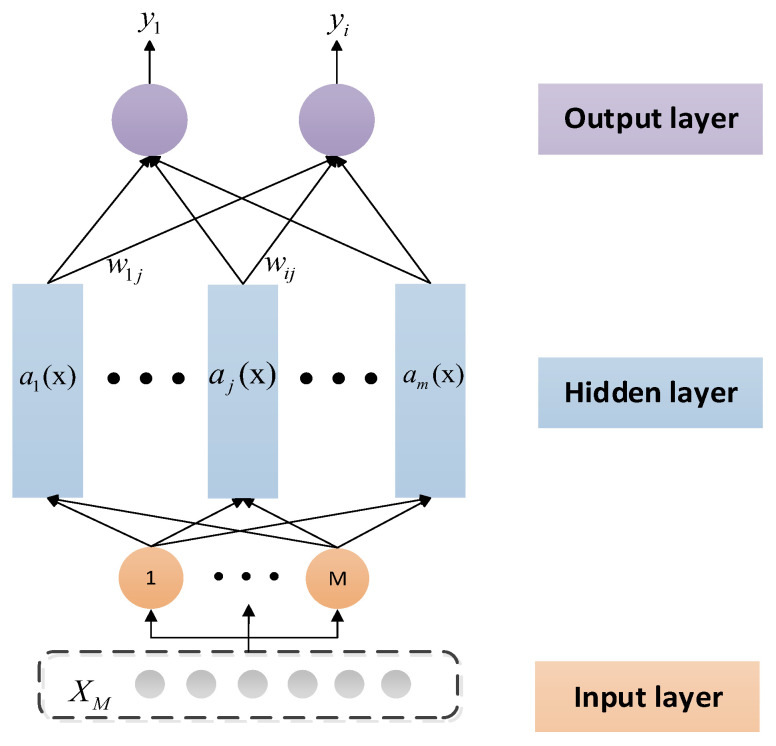
Model of a radial basis function neural network.

To establish an RBF neural network model, an RBF is introduced into the hidden layer of the neural network. The RBF NN structure is shown in Figure 5, which is similar to the other forward neural networks. The RBF neural network has three layers: the input layer, the hidden layer, and the output layer. The input layer consists of signal source nodes. The number of elements in the hidden layer is determined according to the specific problem, and the hidden layer centre transform function is a radial basis function. The output layer is the response to the input mode.

Here, the Gaussian basis function is chosen as the transfer function of the RBF neurons, which is shown in Equation (7):

(7) $$a_{j}(x)=\psi_{j}(\left\|x-c_{j}\right\|/\sigma_{j})=e^{-\frac{{\left\|x-c_{j}\right\|}^{2}}{2\sigma_{j}^{2}}}$$

The output of the RBF NN is shown in Equation (8):

(8) $$y_{i}=\sum_{j=1}^{m}w_{ij}\alpha_{j}(x),i=1,2,\cdot \cdot \cdot ,r$$

where *W_ij_* is the connection weight between the *ith* hidden layer node and the *jth* output layer node. The learning algorithm is shown in Equation (9):

(9) $$w_{ij}(l+1)=w_{ij}(l)+\beta [y_{i}^{d}-y_{i}(l)]\alpha_{j}(x)/\alpha^{T}(x)\alpha (x)$$

where *β* is the learning rate. When *β* falls within the range of 0 to 2, the algorithm offers good convergence performance.

During the training process of the RBF NN, the local amplification of the RBF neural network occurs. If *x* is far from *c_j_*, the output of α_j_(*x*) is also nearly 0 when passing through the linear nerve of the second layer. When *x* and *c_j_* become very close to 0, the output of the layer is almost equal to 1, and the output value is almost equal to the weight value on the second layer when passing through the second layer

The RBF neural network has the characteristics of a simple structure, fast operation speed, and local function approximation. In general, the more hidden layer neuron nodes of the RBF neural network, the stronger the computing power and mapping ability are and the better the function is. The approximation ability can approach a complex function curve with arbitrary precision.

However, the performance of the RBF NN model depends mainly on the length of the training sample set and the expansion constant of the radial basis function (breadth *σ_j_*). The length of the training sample set affects the performance of the network and the real-time performance of the training directly; if the expansion constant, *σ_j_*, is too small, the network will over-fit, and, conversely, if it is too large, the network will not have the ability to fit. Therefore, genetic algorithms are introduced to solve this problem.

**Figure 6:**
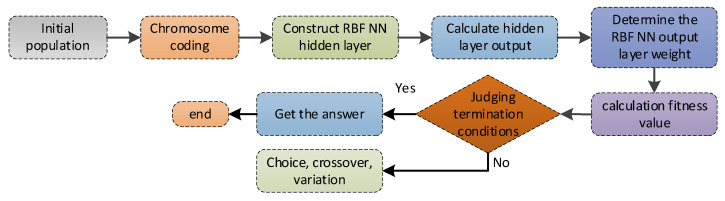
The process of the RBF NN based on a GA (RBF NN+GA).

The genetic algorithm is an adaptive global probability search algorithm that simulates the inheritance and survival of creatures in their natural environment. The algorithm mainly includes five basic steps: coding, initial population generation, fitness setting function, genetic operator design, and operational parameter determination. The process of a GA is shown in Figure 6. In combining the GA with the RBF neural network, use the GA to solve the parameter selection problem of the RBF NN model. The specific algorithm is as follows: set the maximum algebra, *G_MAX_*, of the genetics and combine each body in the population as RBF NN training parameters to find the corresponding output error. After training the algebra maximum, stop the calculation.

The Kalman filter algorithm is an optimal estimation of the autoregressive data processing algorithm. It is widely used in the elimination of gyro noise because of its high estimation accuracy, fast convergence speed, strong adaptability, and simple calculation. Since the original data contain the influence of noise, the optimal choice of data processing can be regarded as a filtering process. The Kalman filtering process can be approximated as the following five equations:

(10) $${\hat{X}}_{k/k-1}=\Phi_{k,k-1}{\hat{X}}_{k-1}$$

(11) $${\hat{X}}_{k}={\hat{X}}_{k/k-1}+K_{k}(Z_{k}-H_{k}{\hat{X}}_{k/k-1})$$

(12) $$K_{k}=P_{k/k-1}H_{k}^{T}{\left(H_{k}P_{k/k-1}H_{k}^{T}+R_{}\right)}^{-1}$$

(13) $$P_{k/k-1}=\Phi_{k,k-1}P_{k-1}\Phi_{k,k-1}^{T}+\Gamma_{k-1}Q_{}\Gamma_{k-1}^{T}$$

(14) $$P_{k}=(I-K_{k}H_{k})P_{k/k-1}$$

where *H_k_* is a measurement matrix, *P_k/k−_*_1_ is a matrix of the prior estimate error covariance, *P_k_* is a matrix of the estimation error variance, *K_k_* is a filter gain matrix, *R* is the covariance of the measurement noise, and *Q* is the covariance of the process noise. According to the above equations, it can be found that the state estimation of ${\hat{X}}_{k}(k=1,2,\cdot \cdot \cdot )$ at time point *k* can be calculated by the measurement vector of *Z_k_* at time point *k* if the initial value of ${\hat{X}}_{0}$ and *P_0_* are determined.

**Figure 7:**
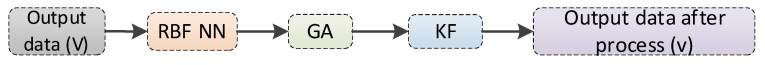
The process of RBF NN+GA+KF.

In this section, the RBF NN, GA, and KF are combined, and the RBF NN+GA+KF fusion algorithm is proposed. The algorithm has the advantages of online real-time use, training capability, and fast learning. The fusion algorithm of RBF NN+GA+KF is shown in Figure 7.

### 3.3. RBF NN+GA+KF Denoising Based on SE-EMD

**Figure 8:**
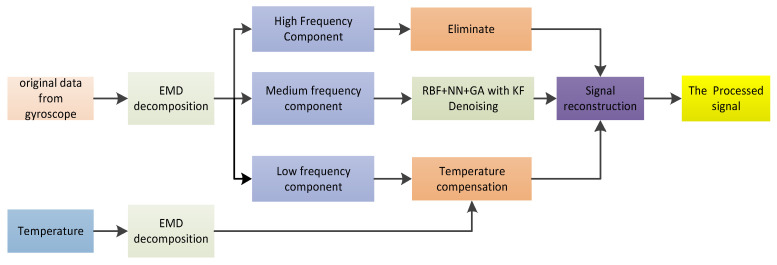
The process of RBF NN+GA+KF denoising based on SE-EMD.

After being decomposed by EMD, the original data are decomposed into multiple IMF components. Each IMF component is classified by using the sample entropy (SE). Generally, these IMF components can be divided into three groups: the noise component, the mixture component, and the drift component. The noise has the characteristics of high frequency and low drift. The high-frequency noise component of the IMFS can be removed directly. However, the mixed component contains both the useful signal and the equivalent noise signal, so the RBF NN+GA+KF is applied to the filtering of this component, which is beneficial for protecting the useful signal of the gyroscope and removing the noise signal effectively. The processing of the drift component can be divided into two steps. First, the output of the temperature sensor is decomposed by EMD to obtain the trend term of the temperature, and then the trend term of the temperature is used to compensate for the drift component of the gyroscope. Finally, the signal reconstruction of the MEMS gyroscope is performed by using the mixed component after denoising and the drift component after compensation by the trend term of the temperature. The specific flow chart is shown in Figure 8.

## 4. Temperature Experiment Proposal

**Figure 9:**
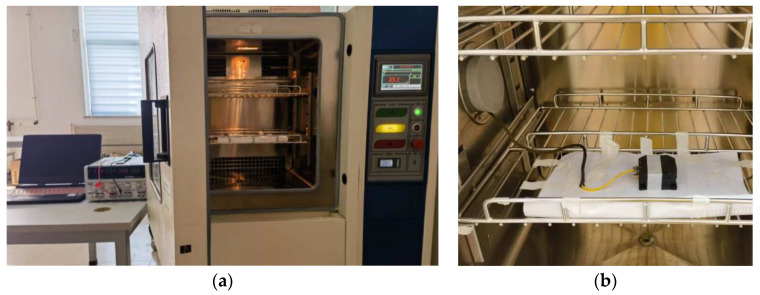
(**a**) Temperature experiment equipment. (**b**) Inside the oven.

Through temperature experiments, the temperature characteristics of the MEMS gyroscope were tested. In the temperature experiment, the temperature-controlled oven can accurately provide a temperature range of −40 °C to +150 °C, as shown in Figure 9. The gyroscope output is collected by dedicated data acquisition software. Power is provided by a Gwinstek GPS-4303C DC power supply. The real-time temperature in the gyroscope metal casing is obtained by a temperature sensor, whose value is synchronised with the gyroscope output. The indoor temperature is 25 °C.

**Figure 10:**
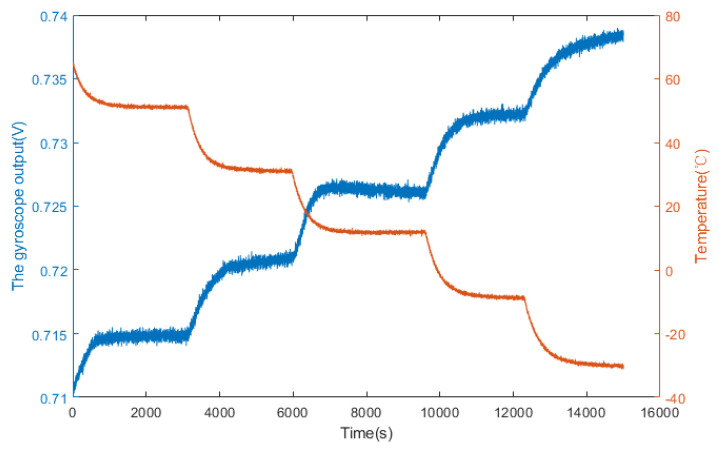
Temperature experiment results in a temperature-controlled oven.

Firstly, the gyroscope is fixed to the static plane to avoid the influence of motion. Secondly, the output lines are connected to the Gwinstek DC power supply and the laptop. Then, the oven temperature is set at −30 °C to +60 °C. Finally, the power is switched on, and the original output signal of the gyroscope is recorded. The data collection process is continuous, and the temperature experiment results are shown in Figure 10. It can be seen that the gyroscope output changes more obviously when the temperature changes. This phenomenon indicates that the temperature affects the precision of the gyroscope output seriously. Therefore, it is necessary to establish a temperature drift model of the MEMS gyroscope.

## 5. Verification and Analysis

**Figure 11:**
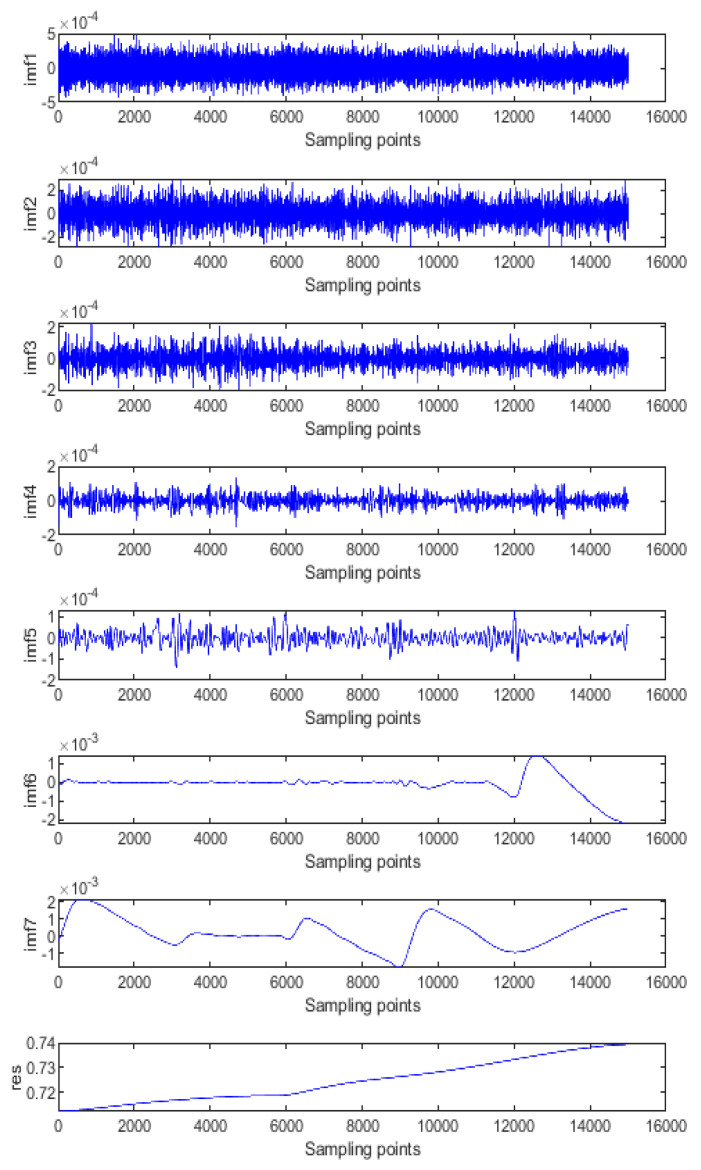
IMFs decomposed by EMD (gyroscope output).

**Figure 12:**
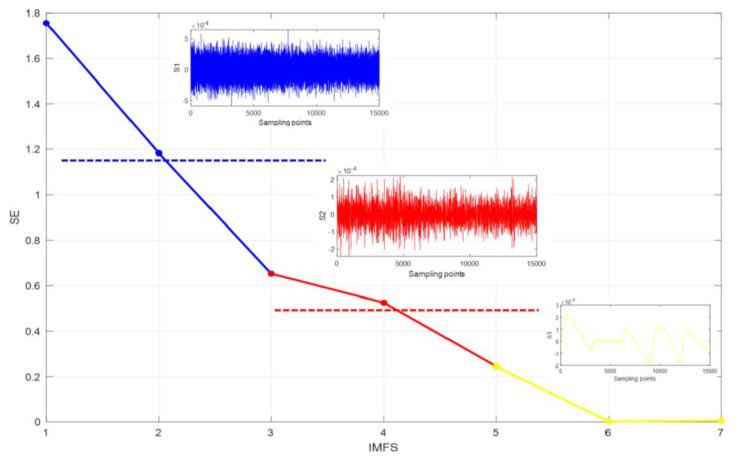
Feature component extraction based on the SE.

**Figure 13:**
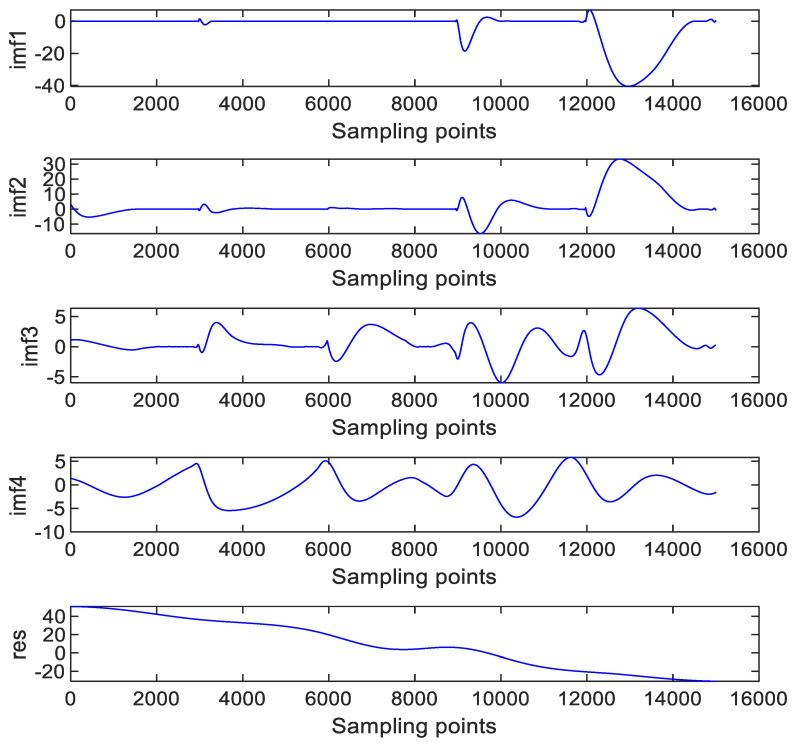
IMFs decomposed by EMD (temperature).

Figure 11 shows the results of the EMD processing of the gyroscope temperature drift error, which is decomposed into 7 IMF components and a residual component. These IMF components are characterised by non-stationary and complex components. For the convenience of processing, the sample entropy (SE) method is used to classify the IMF components of each layer, which can reduce the cumulative error when processing a single IMF reconstruction. Figure 12 shows the feature extraction based on the SE. It can be seen from Figure 13 that these IMF components can be roughly divided into three groups: IMF1, IMF2 as a group; IMF3, IMF4 as a group; and IMF5 to IMF7 as a group. These three groups are the three signal components mentioned above: the noise component (*S1*), the mixed component (*S2*), and the drift component (*S3*). The three signals are processed separately below.

**Figure 14:**
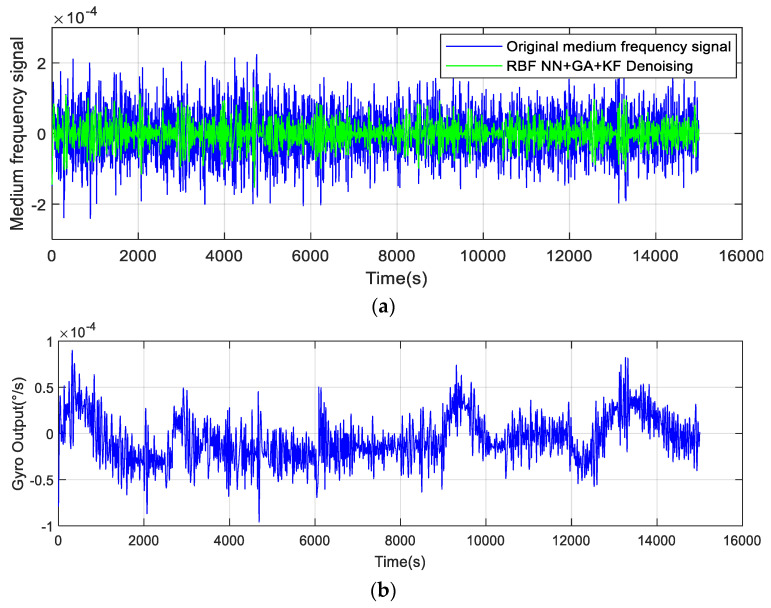
(**a**) Mixed component after denoising, (**b**) The final reconstructed signal.

According to the temperature error handling method proposed in Figure 8, first, the noise component (*S*1) is removed due to the high-frequency and low-drift characteristics of the noise, and the mixed component (*S*2) is denoised by the RBF NN+GA+KF algorithm. The denoised results are shown in Figure 14a. Second, after the temperature sensor output signal is decomposed by EMD, as shown in Figure 13, the temperature trend is obtained. Then, the temperature component is used to compensate for the drift component of the gyroscope output. Finally, the *S*2 and *S*3 component signals after processing are reconstructed, and the reconstruction results are shown in Figure 14b.

It can be seen from Figure 14 that the EMD improved algorithm proposed in this paper can effectively eliminate the error caused by temperature drift.

**Figure 15:**
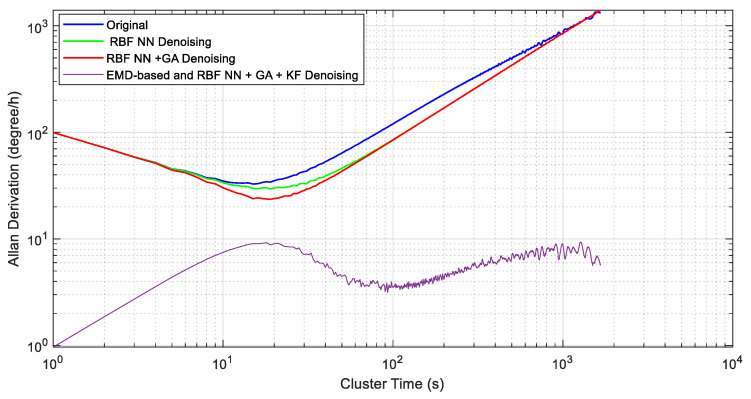
Allan variance analysis of the gyroscope’s output after error processing.

To compare the temperature compensation results quantitatively, the Allen analysis of variance was introduced. The Allen analysis is an analysis method that is based on the time domain. The main feature of the Allan variance method is that it can easily characterise and identify various error sources and their contribution to the overall noise statistics, and it also has the advantages of easy calculation and easy separation. Figure 15 shows the different compensation results for the Allan standard deviation curve, and Table 2 gives the quantitative results. It can be concluded from Table 2 that the proposed EMD-based RBF NN+GA+KF fusion method has the best performance, and the bias stability is increased from 34.66°/h to 3.589°/h. The angular random walk is reduced from 99.608°/h/Hz^1/2^ to 0.967814°/h/Hz^1/2^.

## 6. Conclusions

This paper studies the design and analysis of a new four-mass vibration MEMS gyroscope (FMVMG). The temperature compensation algorithm of the MEMS gyroscope is also studied in detail. First, the structure is simulated by finite element analysis software. The simulation results show that the FMVMG has two working modes: a driving mode and a sensing mode. The resonant frequency of the driving mode is 9.6096 kHz, the resonant frequency of the sensing mode is 9.6154 kHz, and the frequency interval between those two modes is 5.8 Hz. In the temperature compensation, a new fusion algorithm based on empirical mode decomposition (EMD) and combining a radial basis function neural network (RBF NN), genetic algorithm (GA), and Kalman filter (KF), is proposed. First, the gyroscope output is decomposed into three components by EMD and the sample entropy (SE), which are the noise component, the mixed component, and the drift component. Second, to process these three components, the specific steps are as follows: the high-frequency noise component is directly removed, and the RBF NN+GA+KF algorithms are used to denoise the mixed components. Third, the trend component of the temperature sensor output is used to compensate for the drift component of the MEMS gyroscope. Finally, the signal is reconstructed to obtain the final compensated signal. The results show that this method not only removes the noise signal of the gyroscope effectively but also protects the effective signal of the gyroscope. Finally, the Allan variance coefficient shows the comparison of the method with the original data. The data show that if the gyroscope output is compensated by the EMD and RBF NN + GA with the KF fusion method, the bias stability is increased from 34.66°/h to 3.589°/h. The angular random walk is reduced from 99.608°/h/Hz^1/2^ to 0.967814°/h/Hz^1/2^.

## Figures and Tables

**Table 1:** MEMS gyroscope structure mechanical values.

Parameter	Value
Elastic modulus (*E*)	169 GPa
Poisson’s ratio (*µ*)	0.27
Density (*ρ*)	2328.3 kg/m³
Prototype length	3015 μm
Prototype width	2331 μm
Prototype height	60 μm

**Table 2:** Allan variance analysis.

Denoising						Temperature Compensation	
Original Data		RBF NN		RBF NN+GA		RBF NN+GA+KF	
B(°/h)	N(°/h/Hz^1/2^)	B(°/h)	N(°/h/Hz^1/2^)	B(°/h)	N(°/h/Hz^1/2^)	B(°/h)	N(°/h/Hz^1/2^)
34.66	99.608	28.42	99.6037	23.65	99.6011	3.589	0.967814

## Data Availability

Not applicable.
